# Supplementary figures and images for: Efficacy and safety of low-molecular-weight-heparin plus citrate in nephrotic syndrome during continuous kidney replacement therapy: retrospective study
Source: PeerJ. 2025 Feb 25;13:e18919. doi: 10.7717/peerj.18919 (PMC11869884; doi:10.7717/peerj.18919)

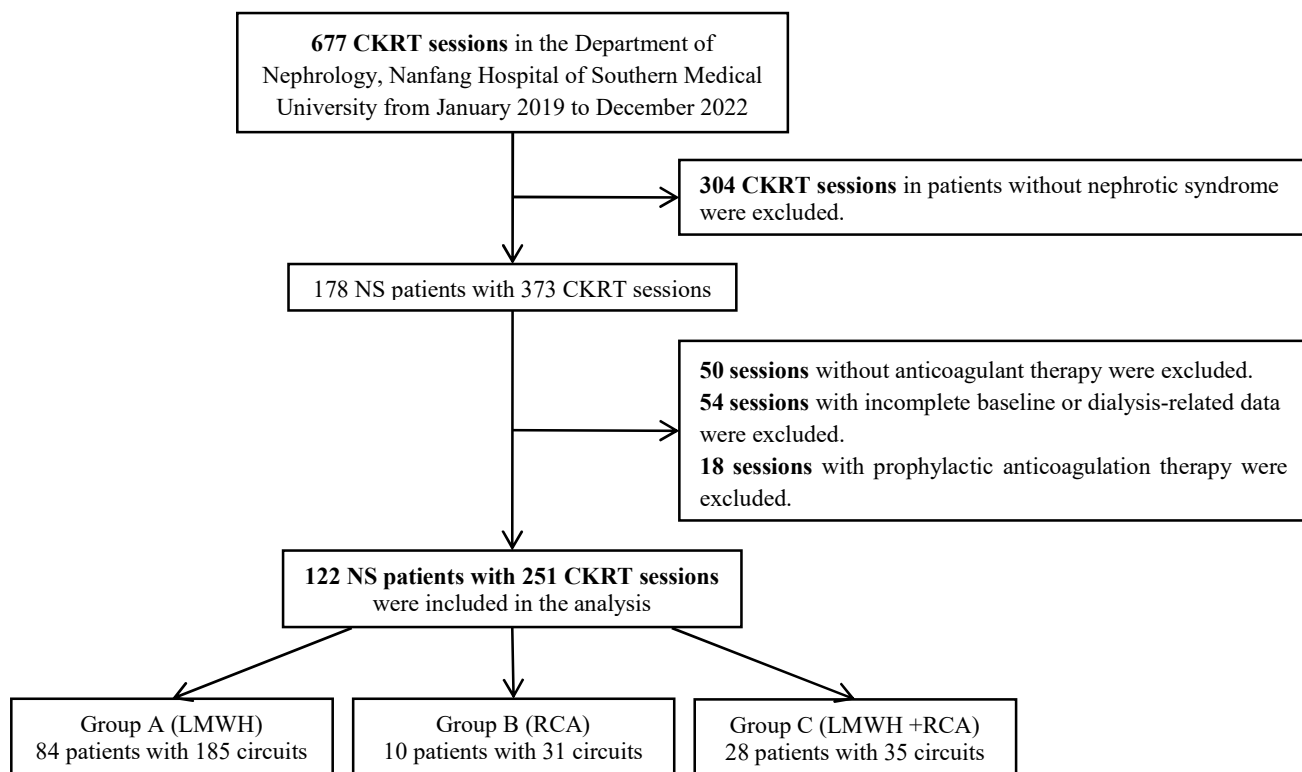

Supplement: Supplemental Information 1 [file peerj-13-18919-s001.pdf]
