# Supplementary material for: Efficacy and safety of low-molecular-weight-heparin plus citrate in nephrotic syndrome during continuous kidney replacement therapy: retrospective study
Source: PeerJ. 2025 Feb 25;13:e18919. doi: 10.7717/peerj.18919 (PMC11869884; doi:10.7717/peerj.18919)
Supplement: Supplemental Information 2 [file peerj-13-18919-s002.docx]

**Supplementary Files**

| **Table S1- Correlation between anticoagulation therapy and filter clotting stratified by subgroup analysis.** | | | | | | | | | | | | | |
| --- | --- | --- | --- | --- | --- | --- | --- | --- | --- | --- | --- | --- | --- |
| **Subgroup** |  | **Group C**  **(LMWH+RCA)** | |  |  | **Group A**  **(LMWH)** | |  |  | **Adjusted ^a^** | | |  |
|  | Total patients | | Circuit clotting | | Total patients | | Circuit clotting | | OR | | 95%CI | P value | |
| **Sex** |  | | | | | | | | | | | | |
| Female | 9 | | 1 | | 52 | | 7 | | 2.195 | | 0.084-57.039 | 0.636 | |
| Male | 26 | | 1 | | 133 | | 45 | | 0.067 | | 0.008-0.575 | 0.014 | |
| **Age** |  | | | | | | | | | | | | |
| ＜60 y | 26 | | 2 | | 110 | | 34 | | 0.130 | | 0.025-0.663 | 0.014 | |
| ≥ 60 y | 9 | | 0 | | 75 | | 18 | | 0.000 | | / | 0.999 | |
| **BMI** |  | | | | | | | | | | | | |
| ＜24.0 kg/m^2^ | 5 | | 1 | | 34 | | 9 | | 0.404 | | 0.039-4.129 | 0.445 | |
| ≥ 24.0 kg/m^2^ | 23 | | 1 | | 103 | | 35 | | 0.064 | | 0.007-0.552 | 0.012 | |
| **ALB** |  | | | | | | | | | | | | |
| ＜20 g/L | 20 | | 2 | | 93 | | 36 | | 0.230 | | 0.041-1.287 | 0.094 | |
| ≥ 20 g/L | 15 | | 0 | | 92 | | 16 | | 0.000 | | / | 0.998 | |
| **eGFR** |  | | | | | | | | | | | | |
| ≤ 30 ml/min·1.73m^2^ | 20 | | 1 | | 107 | | 20 | | 0.100 | | 0.007-1.341 | 0.082 | |
| ＞30 ml/min·1.73m^2^ | 15 | | 1 | | 78 | | 32 | | 0.101 | | 0.011-0.928 | 0.043 | |
| **HGB** |  | | | | | | | | | | | | |
| ＜90 g/L | 14 | | 0 | | 63 | | 8 | | 0.000 | | / | 0.999 | |
| ≥ 90 g/L | 21 | | 2 | | 122 | | 44 | | 0.138 | | 0.027-0.715 | 0.018 | |
| Abbreviations: LMWH = Low molecular weight heparin, BMI = body mass index, ALB = albumin, HGB = hemoglobin, RCA=regional citrate anticoagulation.  ^a^ adjusted for age, gender, baseline BMI, serum albumin, eGFR (CKD-EPI), hemoglobin and platelet. | | | | | | | | | | | | | |

| **Table S2-1. Efficacy of different anticoagulation therapy excluding patients with oral anticoagulants.** | | | | |
| --- | --- | --- | --- | --- |
|  | **Group A**  (LMWH, 185) | **Group B**  (Citrate, 35) | **Group C**  (LMWH + Citrate, 31) | **P value** |
| **Primary outcomes** |  |  |  |  |
| Filter clotting, No. (%) | 52（28.1） | 3（8.6） | 2（6.5） | **＜0.01** |
| Filter lifespan ^a^, median (IQR), h | 16.0（8.0-20.3） | 16.0（10.0-20.0） | 22.5（17.0-24.0） | **＜0.01** |
| **Secondary outcomes** |  |  |  |  |
| Delivered CKRT dose, median (IQR), ml/h·kg | 29.4（26.4-32.3） | 28.9（25.6-31.2） | 28.0（21.8-29.7） | **＜0.01** |
| Initial infusion rate of Citrate, median (IQR) ml/h | / | 180.0（180.0-180.0） | 160.0（160.0-160.0） | **＜0.01** |
| Daily dose of LMWH ^b^, median (IQR), AXaIU | 12000（9300-13322） | / | 8727 (8000-10682） | **＜0.01** |
| Ultrafiltration, median (IQR), ml/h | 323.4（255.0-376.2） | 232.6（263.1-377.6） | 351.7（221.9-399.7） | **＜0.01** |
| Abbreviations: LMWH= Low molecular weight heparin., CKRT= continuous kidney replacement therapy  ^a^ Filter lifespan was defined as the interval from initiation to a non-elective cessation of the circuit, necessitated by filter clotting or reaching the conclusion of treatment in the absence of clotting  ^b^ Daily dose was calculated by averaging the cumulative LMWH dose of each circuit by the actual treatment time of each cycle, and then multiplied it by 24 hours. | | | | |

| **Table S2-2. Efficacy of different anticoagulation therapy including patients with prophylactic anticoagulation** | | | | |
| --- | --- | --- | --- | --- |
|  | **Group A**  (LMWH, 185) | **Group B**  (Citrate, 35) | **Group C**  (LMWH + Citrate, 31) | **P value** |
| **Primary outcomes** |  |  |  |  |
| Filter clotting, No. (%) | 56（28.3） | 4（10.8） | 2（5.9） | **＜0.01** |
| Filter lifespan ^a^, median (IQR), h | 16.0（8.0-20.0） | 16.0（10.0-20） | 22.0（16.0-24.0） | **＜0.01** |
| **Secondary outcomes** |  |  |  |  |
| Delivered CKRT dose, median (IQR), ml/h·kg | 28.9（25.8-32.3） | 28.9（28.9-31.3） | 28.0（21.9-29.5） | **＜0.01** |
| Initial infusion rate of Citrate, median (IQR) ml/h | / | 180.0（180.0-180.0） | 160.0（160.0-160.0） | **＜0.01** |
| Daily dose of LMWH ^b^, median (IQR), AXaIU | 12000（9600-13714） | / | 8864（8864-11182） | **＜0.01** |
| Ultrafiltration, median (IQR), ml/h | 323.4（258.5-379.6） | 331.7（331.7-377.5） | 336.1（287.0-396.9） | **＜0.01** |
| Abbreviations: LMWH= Low molecular weight heparin., CKRT= continuous kidney replacement therapy  ^a^ Filter lifespan was defined as the interval from initiation to a non-elective cessation of the circuit, necessitated by filter clotting or reaching the conclusion of treatment in the absence of clotting  ^b^ Daily dose was calculated by averaging the cumulative LMWH dose of each circuit by the actual treatment time of each cycle, and then multiplied it by 24 hours. | | | | |

| **Table S3 - The cumulative and average dose of LMWH and average infusion rate of citrate between groups.** | | | | |
| --- | --- | --- | --- | --- |
|  | **Group A**  (LMWH, 185) | **Group B**  (Citrate, 31) | **Group C**  (LMWH + Citrate, 35) | **P value** |
| Daily dose of LMWH ^*^, median (IQR), ml/24h | 1.2（1.0-1.7） | / | 0.9（0.8-1.2） | **＜0.01** |
| Average dose of LMWH, median (IQR), ml/h | 0.05（0.04-0.06） | / | 0.04（0.03-0.05） | **＜0.01** |
| Daily dose of LMWH ^*^, median (IQR), AXaIU/24h | 12000 (10286-16500) | / | 9000 (8000-12000) | **＜0.01** |
| Average dose of LMWH, median (IQR), AXaIU/h | 500 (429-688) | / | 375 (333-500) | **＜0.01** |
| Initial infusion rate of Citrate, median (IQR), ml/h | / | 180（180-180） | 160（160-160） | **＜0.01** |
| * Averaging the cumulative LMWH dose of each circuit by the actual treatment time of each cycle, and then multiplied it by 24 hours to get the daily dose of LMWH  Abbreviations: LMWH = low molecule weight heparin. | | | | |

| **Table S4 - The percentage of different dialysis modalities in different groups.** | | | |
| --- | --- | --- | --- |
| **CKRT mode** | **Group A**  (LMWH, 185) | **Group B**  (Citrate, 31) | **Group C**  (LMWH + Citrate, 35) |
| CVVH, No. (%) | 3（1.6） | 0 | 1（2.9） |
| CVVHD, No. (%) | 123（66.5） | 13（41.9） | 32（91.4） |
| CVVHDF, No. (%) | 18（9.7） | 18（58.1） | 1（2.9） |
| SCUF, No. (%) | 41（22.2） | 0 | 1（2.9） |
| Abbreviations: CVVHD= continuous venovenous haemodialysis, CVVH= continuous venovenous haemofiltration, CVVHDF= continuous venovenous haemodiafiltration, SCUF= slow continuous ultrafiltration. | | | |

| **Table S5 - Efficacy of different anticoagulation therapy in** **CVVHD.** | | | | |
| --- | --- | --- | --- | --- |
|  | **Group A**  (LMWH, 123) | **Group B**  (Citrate, 13) | **Group C**  (LMWH + Citrate, 32) | **P value** |
| Filter clotting, No. (%) | 27（22.0） | 2（15.4） | 1（3.2） | **＜0.01** |
| Filter lifespan, median (IQR), h | 16.0（8.0-19.0） | 13.0（8.0-16.0） | 22.5（16.0-24.0） | **＜0.01** |
| Abbreviations: LMWH = low molecule weight heparin. | | | | |

| **Table-S6 - Different blood flow rate and initial rate of citrate between groups.** | | | |
| --- | --- | --- | --- |
|  | **Group A**  (LMWH, 185) | **Group B**  (Citrate, 31) | **Group C**  (LMWH + Citrate, 35) |
| **Blood flow** |  |  |  |
| ＜150ml/min, No. (%) | 31 (16.8) | 1 (3.2) | 2 (5.7) |
| 150ml/min, No. (%) | 126 (68.1) | 28 (90.3) | 33 (94.3) |
| ＞150ml/min, No. (%) | 28 (15.1) | 2 (6.5) | 0 |
| **Initial infusion rate of citrate** |  |  |  |
| 160ml/h, No. (%) | / | 0 | 31 (88.6) |
| 180ml/h, No. (%) | / | 28 (90.3) | 4 (11.4) |
| 200ml/h, No. (%) | / | 3 (9.7) | 0 |
| Abbreviations: LMWH = low molecule weight heparin. | | | |

| **Table S7 - The primary outcomes in CKRT with blood flow of 150ml/min** | | | | |
| --- | --- | --- | --- | --- |
|  | **Group A**  (LMWH, 126) | **Group B**  (Citrate, 28) | **Group C**  (LMWH + Citrate, 33) | **P value** |
| Filter clotting, No. (%) | 33（26.2） | 3（10.7） | 1（3.0） | **＜0.01** |
| Filter lifespan, median (IQR), h | 15.0（8.0-20.0） | 15.5（8.3-16.0） | 22.0（16.0-24.0） | **＜0.01** |
| Abbreviations: LMWH = low molecule weight heparin. | | | | |

| **Table S8 - The pathological classification of renal biopsy in 94 patients.** | | | |
| --- | --- | --- | --- |
| **Pathological classification** | **Group A**  (LMWH, 62) | **Group B**  (RCA, 10) | **Group C**  (LMWH+RCA, 22) |
| **Primary** | 45 | 5 | 17 |
| MCD | 16 | 3 | 7 |
| MN | 14 | 2 | 5 |
| FSGS | 14 | 1 | 4 |
| MPGN | 2 | 0 | 1 |
| **Secondary** | 16 | 4 | 5 |
| Lupus nephritis | 8 | 3 | 3 |
| Diabetic nephropathy | 7 | 1 | 2 |
| IgA vasculitis | 1 | 0 | 0 |
| Abbreviations: MCD = Minimal change disease, MN = Membranous nephropathy, FSGS = Focal segmental glomerulosclerosis, MPGN = membranoproliferative glomerulonephritis, LMWH = low molecule weight heparin, RCA = regional citrate anticoagulation. | | | |

| **Table S9 - Primary outcomes of different anticoagulation therapy after excluding patient with prostate cancer.** | | | | |
| --- | --- | --- | --- | --- |
|  | **Group A**  (LMWH, 182) | **Group B**  (Citrate, 31) | **Group C**  (LMWH + Citrate, 35) | **P value** |
| Filter clotting, No. (%) | 52（28.6） | 3（9.7） | 2（5.7） | **＜0.01** |
| Filter lifespan, median (IQR), h | 15.0（8.0-19.0） | 15.0（8.0-16.0） | 22.0（16.0-24.0） | **＜0.01** |
| Abbreviations: LMWH = low molecule weight heparin | | | | |

| **Table S10 - Primary outcomes of different anticoagulation therapy after excluding patients with cancer.** | | | | |
| --- | --- | --- | --- | --- |
|  | **Group A**  (LMWH, 173) | **Group B**  (Citrate, 31) | **Group C**  (LMWH + Citrate, 35) | **P value** |
| Filter clotting, No. (%) | 51（29.5） | 3（9.7） | 2（5.7） | **＜0.01** |
| Filter lifespan ^a^, median (IQR), h | 15.0（8.0-19.0） | 15.0（8.0-16.0） | 22.0（16.0-24.0） | **＜0.01** |
| Abbreviations: LMWH = low molecule weight heparin | | | | |

| **Table S11 - The comorbid diseases of patients between three groups.** | | | | |
| --- | --- | --- | --- | --- |
| **Comorbid diseases** | **Group A**  (LMWH, 84) | **Group B**  (RCA, 10) | **Group C**  (LMWH+RCA, 28) | **P value** |
| HTN, No. (%) | 47 (56.0) | 8 (80.0) | 17 (60.7) | 0.336 |
| DM, No. (%) | 24 (28.6) | 3 (30.0) | 14 (50.0) | 0.112 |
| CVDs, No. (%) | 10 (11.9) | 0 | 1 (3.6) | 0.622 |
| HBV, No. (%) | 7 (8.3) | 1 (10.0) | 3 (10.7) | 0.924 |
| Malignant tumor, No. (%) | 4 (4.8) | 0 | 0 | 0.756 |
| Abbreviations: HTN = Hypertension, DM = Diabetes mellitus, CVDs = cardiovascular disease and/or cerebrovascular disease, HBV = hepatitis B viral infection, LMWH = low molecule weight heparin, RCA = regional citrate anticoagulation. | | | | |

| **Table S12 - Efficacy of different anticoagulation therapy after excluding patients ＜ 18 years old.** | | | | |
| --- | --- | --- | --- | --- |
|  | **Group A**  (LMWH, 164) | **Group B**  (Citrate, 31) | **Group C**  (LMWH + Citrate, 34) | **P value** |
| **Primary outcomes** |  |  |  |  |
| Filter clotting, No. (%) | 45（27.4） | 3（9.7） | 1（2.9） | **＜0.01** |
| Filter lifespan ^a^, median (IQR), h | 16.0（8.0-19.0） | 15.0（8.0-16.0） | 22.0（16.9-24.0） | **＜0.01** |
| ^a^ Filer lifespan means the time from the beginning of CKRT treatment to filter clotting or until 24 hours.  Abbreviations: LMWH = low molecule weight heparin. | | | | |
